# Direct RNA sequencing dataset of SMG1 KO mutant Physcomitrella (*Physcomitrium patens*)

**DOI:** 10.1016/j.dib.2020.106602

**Published:** 2020-11-29

**Authors:** Andrey Knyazev, Anna Glushkevich, Igor Fesenko

**Affiliations:** Shemyakin-Ovchinnikov Institute of Bioorganic Chemistry of the Russian Academy of Sciences, 16/10, Ulitsa Miklukho-Maklaya, Moscow, 117997, Russian Federation

**Keywords:** Transcriptomics, Nonsense-mediated decay, Direct RNA sequencing, Physcomitrella (*Physcomitrium patens*), SMG1 knockout

## Abstract

Nonsense-mediated mRNA decay (NMD) is a system that controls the quality of mRNA transcripts in eukaryotes by degradation of aberrant transcripts in a pioneer round of translation. In mammals, NMD targets one-third of mutated, disease-causing mRNAs and ∼10% of unmutated mRNAs, facilitating appropriate cellular responses to environmental changes [1]. In plants, NMD plays an important role in development and regulating abiotic and biotic stress responses [2]. The transcripts with premature termination codons (PTCs), upstream ORFs or long 3′-UTRs can be targeted to NMD. It was shown that alternative splicing plays a crucial role in regulation of NMD triggering, for example, by the introduction of a PTC in transcripts. Therefore, the correct identification of mRNA isoforms is a key step in the study of the principles of regulation of the cell transcriptome by the NMD pathway. Here, we performed long-read sequencing of Physcomitrella (*Physcomitrium patens*) mutant *smg1*Δ line 2 native transcriptome by Oxford Nanopore Technology (ONT). The *smg1*Δ is a knockout (KO) mutant deficient in SMG1 kinase is a key component of NMD system in plants and animals [3]. RNA was isolated with Trizol from 5 day old protonemata and sequenced using kit SQK-RNA002, flow cells FLO-MIN106 and a MinION device (Oxford Nanopore Technologies Ltd., UK (ONT)) in three biological repeats. Basecalling was performed with Guppy v.4.0.15. The presented transcriptomes give advantages in the identification and functional characterization of RNA transcripts that are direct targets of the Nonsense-mediated mRNA decay system.

## Specifications Table

**Table:** 

Subject	Molecular biology
Specific subject area	Transcriptomics
Type of data	Transcriptome sequences
How data were acquired	Direct RNA sequencing was performed with a MinION (Oxford Nanopore Technologies Ltd., UK (ONT)), R9.4.1 flow cells and Guppy v.4.0.15 basecaller
Data format	Raw reads in FASTQ format
Parameters for data collection	Protonemata of mutant line smg1Δ were grown in 200 ml liquid BCD medium supplemented with 5 mM ammonium tartrate (BCDAT) during a 16-h photoperiod at 250C [4]. After 5 days, protonemata were collected for the analysis. The experiment was performed in three biological replicates.
Description of data collection	RNA was isolated by Trizol and poly(A) was selected using Poly(A)Purist™-MAG. Direct RNA sequencing kit by Oxford Nanopore (SQK-RNA002) was used for library preparations.
Data source location	Shemyakin-Ovchinnikov Institute of Bioorganic Chemistry of the Russian Academy of Sciences Moscow Russia
Data accessibility	Repository name: BioProject Data identification number: PRJNA670829 Direct URL to data: https://www.ncbi.nlm.nih.gov/sra/PRJNA670829

## Value of the Data

• The identification of NMD targets is a challenging task. In this context, the ONT direct RNA sequencing seems to be the ideal technology for the comprehensive and correct identification of all mRNA isoforms in NMD-deficient mutants because of its ability to identify full native transcripts [5]. This is the first dataset that describes native transcriptomes of plants with a disrupted NMD system.
• The moss *P. patens* is a suitable model for studying the NMD pathway in plants [6]. Therefore, the presented dataset can be used for the analysis of transcriptome regulation in eukaryotes.
• Using nanopore sequencing is the main advantage of the reported dataset because of the analysis of native transcripts. Therefore, it might be used for revealing new RNA targets of the NMD system in plants. Using this dataset, one can also correct own RNA-seq data and investigate the principles of plant transcriptome regulation.

## 1 Data Description

**Fig. 1:**
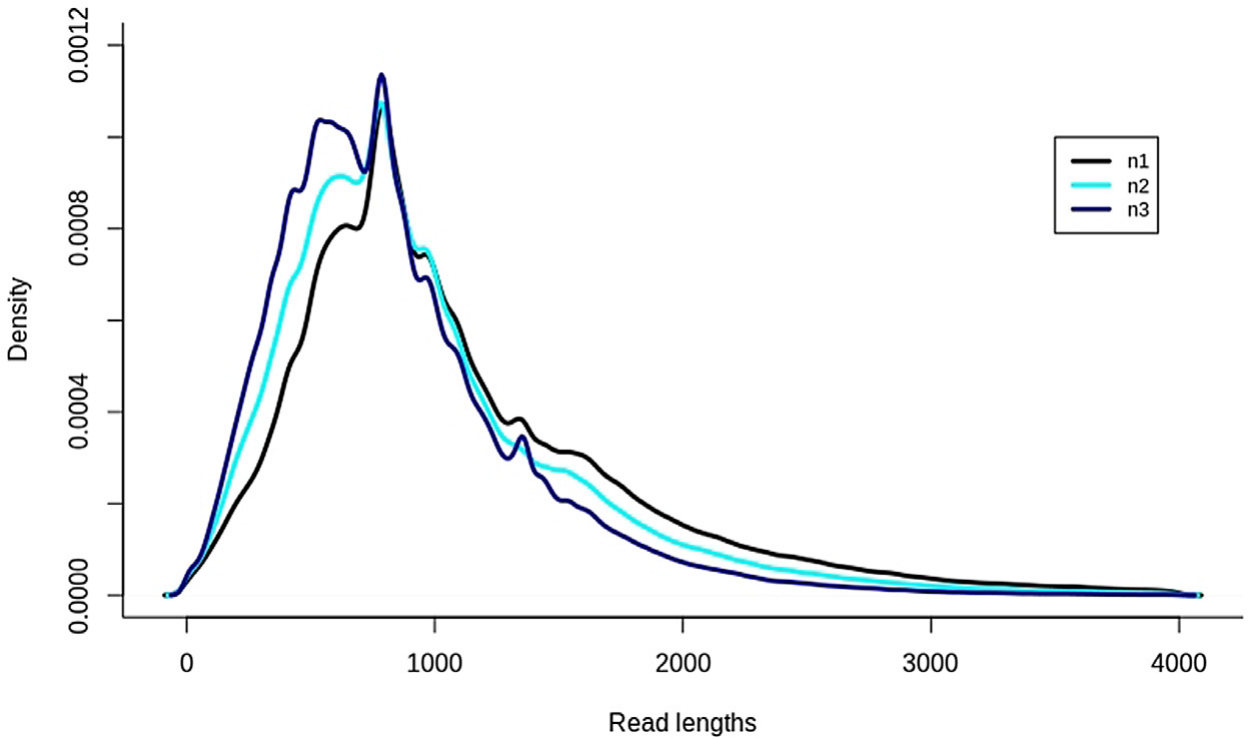
Distributions of read lengths. Line colours represent sample IDs.

The dataset contains data obtained through the sequencing of purified polyadenylated RNAs extracted from the moss Physcomitrella (*Physcomitrium patens*) SMG1 KO mutant [3]*.* The three biological replicates were sequenced with a MinION sequencer (Oxford Nanopore Technologies Ltd., UK (ONT)). Each library was sequenced in an individual flow cell (R9.4.1) during 72 h. Raw data was basecalled to FASTQ with Guppy v.4.0.15. FASTQ files were deposited in NCBI Sequence Read Archive and are accessible through the BioProject: **PRJNA670829**. The main information about runs is shown in Table 1. The reads of the control sample (RNA CS) used in the library preparation (SQK-RNA002) were not filtered. Read quality score is calculated as the mean Phred quality score of all read nucleotides. Default minimum value of quality score for further analysis is 7. More than 87% of reads had a quality score higher than 7, and mean quality score among all reads lay between 9.4 and 10.2. Using minimap2, more than 90% of obtained nanopore reads were mapped to the reference genome, suggesting the high quality of data. The longest read is 51089 nucleotides, and its quality score is higher than 7. The distribution of read lengths is shown in Fig. 1. Only reads with length less than 4000 nucleotides are presented because longer reads are rare.

**Table 1:** Descriptive statistics of sequences submitted to the NCBI Sequence Read Archive (SRA).

ID	Biosample accession no.	Mean Qscore	Total number of reads	Median read length	Reads mapped to reference	Reads mapped to control	Reads with Qscore >= 7	Max length
n1	SAMN16539252	10.1	1514213	932	93.8%	1.35%	94.6%	51089
n2	SAMN16539253	9.4	1321600	830	91.25%	0.45%	87.7%	22715
n3	SAMN16539254	10.2	1895455	759	91.8%	2.68%	94.3%	41623

## 2 Experimental Design, Materials and Methods

SMG1 is the core kinase of the NMD machinery. Several lines with a deleted *SMG1* in the basal land *Physcomitrella patens* subsp. *patens* (“Gransden 2004”, Frieburg) were produced by James P. B. Lloyd [3]. One of these lines, SMG1 KO mutant line 2, was used for direct RNA sequencing by Oxford Nanopore Technology (ONT). Protonemata of the mutant line were grown in 200 ml liquid BCD medium supplemented with 5 mM ammonium tartrate (BCDAT) during a 16 h photoperiod at 25 °C for 5 days [4]. Total RNA from protonemata of three biological repeats was isolated using TRIzol™ Reagent. RNA quality and quantity were evaluated via electrophoresis in an agarose gel with ethidium bromide staining. The precise concentration of total RNA in each sample was measured using a Qubit™ RNA HS Assay Kit, 5–100 ng on a Qubit 3.0 (Invitrogen, US) fluorometer. 100 μg aliquots of total RNA were diluted in 100  μl of nuclease-free water, and poly(A) was selected using Poly(A)Purist™-MAG Purification Kit Invitrogen by Thermo Fisher Scientific. Resulting poly(A) RNA was eluted in nuclease-free water. The Direct RNA sequencing kit by Oxford Nanopore (SQK-RNA002) including the optional reverse transcription step was used to prepare libraries from the poly(A) RNA. 200 ng total library was loaded in FLO-MIN106 (ONT R9.4) flow cells and sequencing on the MinION platform and standard MinKNOW software. We used Guppy 4.0.15 (Oxford Nanopore Technologies) for basecalling direct RNA sequencing data. MinIONQC.R script [7] and Samtools v.1.10 [8] were used to calculate FASTQ quality control statistics. Minimap2 v.2.17 [9] with parameters *-ax splice -uf -k14 -G2k* was used to align reads to Physcomitrella (*Physcomitrium patens*) genome (assembly version v3) with added yeast enolase control sequence.

## Declaration of Competing Interest

The authors declare that they have no known competing financial interests or personal relationships which have, or could be perceived to have, influenced the work reported in this article.
